# Migraine among students of a medical college in western China: a cross-sectional study

**DOI:** 10.1186/s40001-022-00698-9

**Published:** 2022-05-23

**Authors:** Haodi Yang, Shengxiong Pu, Yang Lu, Wenxiu Luo, Jiayu Zhao, Enzhuo Liu, Jiaming Yang, Xinya Luo, Xinyi Tang, Cheng Zeng, Jie Chen, Jiaming Luo

**Affiliations:** 1 Department of Neurology, the Affiliated Hospital of North Sichuan Medical College, Nanchong, 637000 Sichuan Province China; 2 Department of Psychiatry, the Affiliated Hospital of North Sichuan Medical College, Nanchong, 637000 Sichuan Province China; 3 North Sichuan Medical College, Nanchong, 637000 Sichuan Province China; 4 School of Psychitry, North Sichuan Medical College, Nanchong, 637000 Sichaun Province China

**Keywords:** Migraine, College students, Characteristics, Gender, Stress

## Abstract

**Objective:**

As one of the most common primary headaches in clinical practice, migraine affects the learning ability and life quality of college students worldwide, posing a heavy burden on individuals and society. This study aims to investigate the incidence of migraine among Chinese medical college students and to explore its characteristics and typical triggers.

**Method:**

From July 2019 to July 2020, North Sichuan Medical College in Sichuan province, China preliminarily screened migraine cases using ID-Migraine through cluster sampling. College students with positive ID-Migraine results would be included in this study if they were further diagnosed with migraine by neurologists based on ICHD-3. After the ethical review, patients’ personal and headache information would be collected, and the frequency, severity, onset time, and related triggers of migraine would be measured.

**Results:**

The preliminary screening covered 8783 college students. The overall prevalence rate of migraine is 6.57%, 5.90% in men and 6.77% in women. The prevalence rate of migraine is higher for students in the first and second grades (8.01%, 8.05%), and students with a family history of migraine are more likely to suffer from migraine (OR = 1.509, 95% CI 1.060–2.148, *P* = 0.022 < 0.005). Staying up late (*n* = 329, 57.01%), stress (*n* = 319, 55.29%), catch a cold (*n* = 313, 54.25%) and sleep disorders (*n* = 302, 52.34%) are the common triggers.

**Conclusion:**

Migraine is common among college students in North Sichuan Medical College. The incidence is higher among lower grade students, female students, and students with a family history of migraine. Improving sleep quality and reducing stress may be effective in relieving migraines.

## Background

Migraine is a common type of recurring headache disorder in clinical practice. The prevalence rate of migraine is about 10 ~ 18% [1–5] worldwide, ranking third according to the survey of 2010 Global Disease Burden; and the rate for women is two or three times higher than that for men [4, 6, 7]. In addition, the disability rate caused by migraine ranks the seventh globally [1, 8–11], posing a heavy financial burden on individuals and society, which includes direct costs (such as medical expenses) and indirect costs (such as absenteeism, lower productivity, and costs) [12]. Once it attacks, college students will see a decline in their learning ability and life quality, and suffer from physiological and psychological distress [13–15].

Most migraineurs have their first onset around the age of 30, and for college students, this time is even earlier, around the age of 20 [14, 16, 17]. According to a meta-analysis on 34,904 college students conducted by Wang et al. [18] in 2016, the overall prevalence rate for migraine among college students was 16.1%, and that for medical students was about 11 ~ 40% [19]. Frequent attacks of migraine undermine people’s life quality greatly as it leads to the inability to participate in extracurricular activities, the deterioration in learning ability and social capability [15]. Medical students are a group of people who are susceptible to migraines as they need to spend a long time to complete a large number of clinical practice under academic pressure, resulting in a disordered diet and poor sleep quality [4, 12, 14, 20].

The first national survey of primary headache conducted from 2008 to 2009 shows that the prevalence rate of migraine was 9.3% [21]. However, studies on the prevalence, characteristics, and triggers of migraine among college students are insufficient. Moreover, the study was conducted in Harbin City, Heilongjiang Province investigated the prevalence of migraine among college students [2]. In 2016, a cross-sectional study was carried out at Soochow University to analyze and discuss the common triggers of migraine [12]. However, the sample size of college migraine patients involved in the two studies was small, and no further analysis was conducted on certain triggers.

Therefore, this study aims to investigate the prevalence of migraine among college students in North Sichuan Medical College, and further identify its common triggers and investigate some of them.

### Study design

From July 2019 to July 2020, North Sichuan Medical College, Sichuan Province, China, preliminarily screened migraine cases with ID-Migraine and cluster sampling. Personal and headache information of college students with positive ID-Migraine results was collected, and the diagnosis was made with the ICHD-3[22].

### Study population

The subjects of this study are undergraduate students of various medical specialties in North Sichuan Medical College (covering grade 1–4), including Department of Clinical Medicine, Department of Forensic Medicine, Department of Management, Department of Nursing, Department of Basic Medicine, Department of Psychiatry, Department of Anesthesiology, Department of Laboratory Medicine, Department of Stomatology, Department of Bioengineering Medicine, Department of Ophthalmology, Department of Pharmacy, Department of Medical Imaging Technology, Department of Preventive Medicine, Department of Rehabilitation Medicine, Department of Midwifery Medicine and Department of Clinical Medicine of Traditional Chinese and Western Medicine.

Inclusion criteria: 1. Migraine was diagnosed by two neurologists (ICHD-3). 2. No endocrine system, immune system, nervous system, and other serious physical diseases. 3. No history of acute or chronic infection in the past one month. 4. No immunosuppressant or enhancer has been used in at least half a year. 5. No migraine drugs, antidepressants or anxiety drugs were used in at least 2 weeks.

Exclusion criteria: 1. Patients with a secondary headache. 2. Patients with other chronic diseases.

### Data collection

**Fig. 1:**
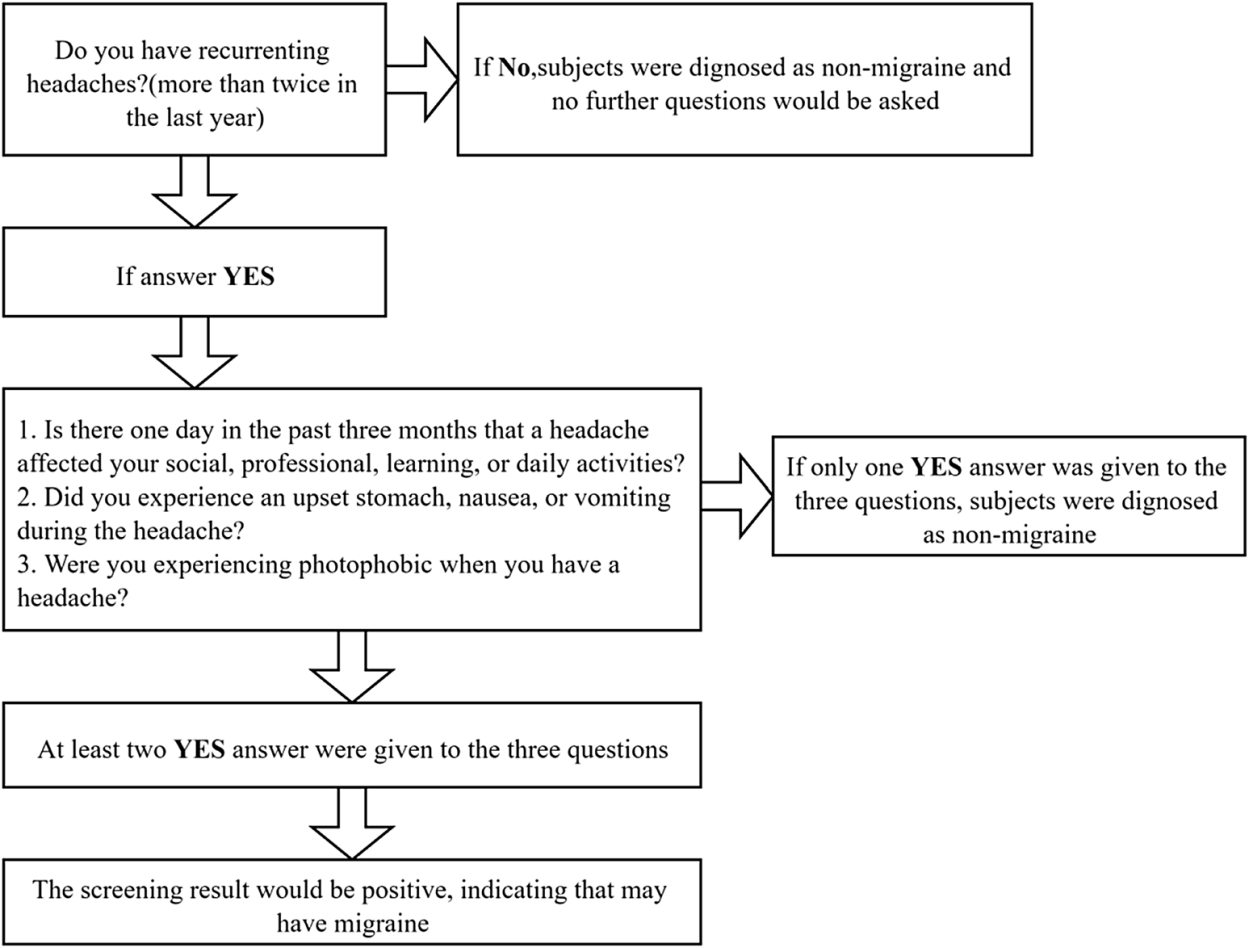
Flow diagram of the preliminary screening

Before the preliminary screening, participants would be asked if they have recurring headaches. If the answer is “yes”, ID-Migraine, a simple and effective tool that is often used to diagnose migraine [2, 4, 23], would be adopted for preliminary screening. Its sensitivity is 0.84 (95% CI 0.75–0.90) and specificity is 0.76 (95% CI 0.69–0.83) [24]. As for the Chinese version, the sensitivity and specificity are 84% and 64%, respectively [25]. Three questions are contained in this tool: 1. Is there one day in the past 3 months that a headache affected your social, professional, learning, or daily activities? 2. Did you experience an upset stomach, nausea, or vomiting during the headache? 3. Were you experiencing photophobia when you have a headache? If two or more of the answers are “yes”, the screening result would be positive, indicating that they may have migraine (see Fig. 1).

Then, personal and headache information was collected. Personal information includes name, gender, nationality, age (year old), height (cm), weight (kg), grade, cost of living (¥/month), smoking history (continuous or cumulative smoking for 6 months or more in one's life) and drinking history (drinking ≥ 500 ml at least once a week in the past 6 months). Headache information includes the triggers of headache, which means the factor that directly leads to migraine attacks for two or more times; headache relief factors, which refers to the factor that could relieve migraine symptoms for two or more times; the age when migraine symptoms first appear or are diagnosed by professional neurologists; and migraine onsets per month (one day would be recorded for onsets lasts less than one day); the duration of every migraine attack, which is calculated from the onset to the end of the symptoms by hours (when the duration exceeds 1 h, the part less than 1 h would be recorded as 1 h); and VAS score, which uses a 10-cm horizontal line drawn in the paper, and the two ends are marked as 0 (painless) and 10 (severe pain). During the test, the patients face the side without a scale and are asked to mark at a certain point to indicate the intensity of pain; while the doctor will record the length from the starting point to the mark, which is the score of pain degree [26].

### Data collection method

The members of the study group (they have been trained in professional knowledge for a long time and have passed relevant tests to ensure that each of them could independently complete one-to-one information collection according to the unified standard) will ask the students with positive preliminary screening results whether they are willing to provide basic personal information and headache information through telephone, e-mail and other ways. During the collection, the information shall be recorded in strict accordance with the unified standards, and security shall be ensured. In case of the failure to collect complete data at that time or omission of information by interviewees, the information shall be completed through telephone or e-mail follow-up. The collected data will be diagnosed by two professional neurologists according to ICHD-3 [22], and the patients diagnosed with migraine will be included in the study.

### Data analysis

The SPSS Statistics version 25 (IBM Corporation, Armonk, NY, USA) was used for data analysis. Continuous variables with normal distributions were described as the mean and SD. Non-normal distributions of continuous data were presented as medians and IQRs. Categorical variables were described as numbers and percentages. Individual sample Student’s *t*-test and ANOVA were used to compare the group differences for normally distributed continuous data. Non-normally distributed continuous data were compared using the Mann–Whitney *U* test. The Chi-square and Fisher’s exact tests were used for categorical data wherever appropriate. Binary logistic regression analysis was adopted to identify the factors associated with migraine status. Differences between groups were considered significant when P was less than 0.05.

## Results

Ultimately, we distributed 9057 ID-Migraine questionnaires for preliminary screening to 17 majors in 4 grades of the whole school and received 8783 valid questionnaires with an effective response rate of 96.97%. The number of questionnaires with positive results was 1037. In the end, a total of 1007 students participated in the further information collection, which includes 220 males (21.85%) and 787 females (78.15%), and the collection rate was 97.11%. After excluding the invalid data, incomplete collection results, the data of headache types that could not be diagnosed by ICHD-3 diagnostic criteria, and the data of patients with major mental diseases, our analysis ultimately included 788 headache patients who were diagnosed with migraine, tension headache and other types of headache.

### Characteristics of participants

**Fig. 2:**
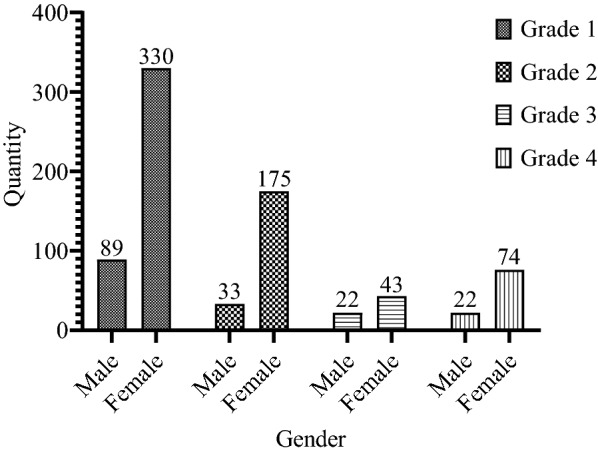
Grade and gender distribution of all participants (N = 788)

All 788 participants (Fig. 2) reporting migraine were within 17–25 years old, with an average age of 19.63 ± 1.66 years, and an average BMI of 20.35 ± 3.07. Among them, 166 (21.06%) were males and 622 (78.94%) were females, with the male–female ratio of 1:3.74, which was similar to that in North Sichuan Medical College. There were 721 (91.50%) students of Han nationality and 67 (8.50%) students of minority nationalities. To be specific, 419 grade one students participated in the study, accounting for 53.17%, followed by 208 (26.40%) grade two, 96 (12.18%) grade four, and 65 (8.25%) grade three students in the descending order. Due to academic needs, the students in grade 5 were receiving a one-year clinical practice in other distant hospitals outside the city, so they did not get involved in this study. Participants with smoking history (*n* = 23, 2.923%) and drinking history (*n* = 39, 4.95%) were not in great numbers. More than half of the students (*n* = 522, 66.24%) had monthly living expenses ranging from RMB 1000 to RMB 1500, while The number of participants with living expenses no less than RMB 1,500 (*n* = 138, 17.52%) was basically the same with that of participants with no more than RMB 1,000 (*n* = 128, 16.24%). A total of 282 (35.79%) participants had family heredity history, while 506 participants showed no family history (64.21%).

Table 1 shows the differences in the demographic characteristics of participants according to gender. There were fewer men than women in each grade, and grade one students took the most active part in the study. More men had a history of smoking and drinking than women, and the average BMI of men was higher than that of women. The rate of migraine patients with a family heredity history was relatively low. All these differences were statistically significant.

**Table 1:** Demographic characteristics of participants according to gender

Characteristics	Total *n* = 788	Male *n* = 166	Female n = 622	*P*
Grade				0.019
Grade 1	419 (53.17%)	89 (53.61%)	330 (53.05%)	
Grade 2	208 (26.40%)	33 (19.87%)	175 (28.13%)	
Grade 3	65 (8.25%)	22 (13.26%)	43 (6.91%)	
Grade 4	96 (12.18%)	22 (13.26%)	74 (11.91%)	
Ethnicity				0.508
Han	721 (91.49%)	154 (92.77%)	567 (91.16)	
Minority	67 (8.51%)	12 (7.23%)	55 (8.84%)	
History of smoking				0.000
Yes	23 (2.92%)	17 (10.24%)	6 (0.96%)	
No	765 (97.08%)	149 (89.76%)	616 (99.04%)	
History of alcohol consumption				0.000
Yes	39 (4.95%)	26 (15.66%)	13 (2.09%)	
No	749 (95.05%)	140 (84.34%)	609 (97.91%)	
Living expenses/per month				0.111
≤ 1000	128 (16.24%)	20 (12.04%)	108 (17.36%)	
1000–1500	522 (66.24%)	121 (72.89%)	401 (64.47%)	
≥ 1500	138 (17.52%)	25 (15.07%)	113 (18.17%)	
Family history				0.009
Yes	282 (35.79%)	45 (27.10%)	237 (38.10%)	
No	506 (64.21%)	121 (72.90%)	385 (61.90%)	
Age, years	19.63 ± 1.66	19.77 ± 1.81	19.60 ± 1.62	0.23
Body mass index, kg/m 2	20.35 ± 3.07	21.79 ± 3.86	19.97 ± 2.70	0.000

Values reported as mean (SD) or n (%) for the total sample and for each gender group where appropriate.

P-values generated from one-way ANOVA or Pearson Chisquare test or Fisher’s exact test where appropriate

### Prevalence of migraine

**Fig. 3:**
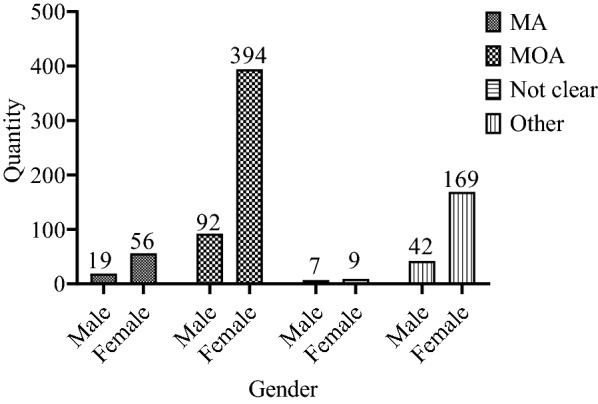
Types of headaches and gender distribution of participants (N = 788)

**Fig. 4:**
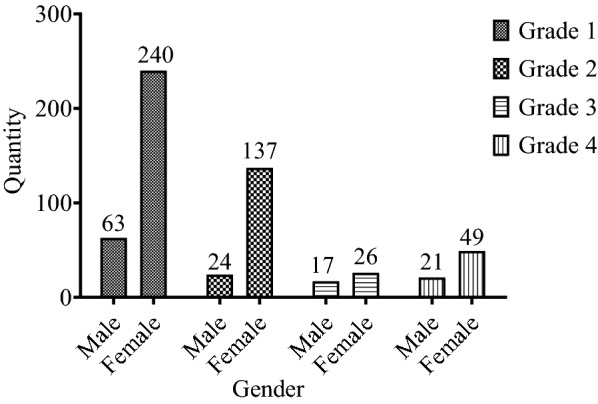
Grade and gender distributions of migraine patients (N = 577)

**Fig. 5:**
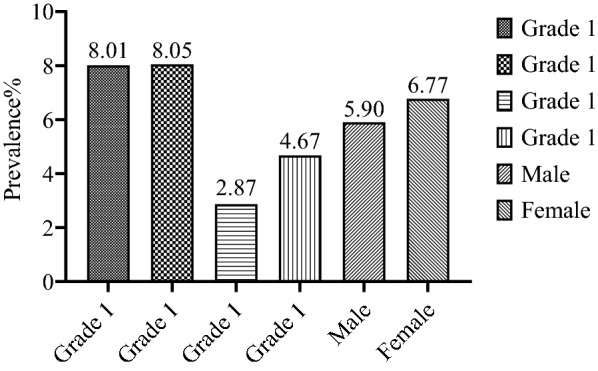
The prevalence of migraine in different grades and the overall prevalence of male and female

Eventually, according to the preliminary positive screening results and the investigation of headache situation and headache history, two neurologists classified and diagnosed the types of headache based on ICHD-3, and excluded those participants who might have migraine or other types of headache that could not be clearly diagnosed, or with major mental diseases. A total of 577 (73.22%) participants were diagnosed with migraine, and the prevalence rate was 6.57% (N_total_ = 8,783), including 76 ones with migraine with aura (N_male_ = 19, 25%, N_female_ = 56, 75%), 486 ones with migraine without aura (N_male_ = 92, 18.93%, N_female_ = 394, 81.07%), and 15 ones with migraine that could not be classified temporarily. A total of 211 participants were diagnosed with tension headache and other types of headache (see Fig. 3). Figure 4 shows the grade and gender distributions of migraine patients, presenting the least number of patients in grade 3 and most patients in grade 1. Figure 5 shows the different prevalence rates of different grades and genders. Table 2 reveals the migraine incidence rate of different student groups in the whole headache population. In this study, monthly living expenses and family heredity history were significantly associated with migraine. Students with average living expenses and family heredity history were found to have a stronger correlation with migraine (*P* < 0.05). By incorporating monthly living expenses and family heredity history of migraine in the logistic regression analysis (Table 3), it is shown that monthly living expenses (RMB 1,000–15,00, OR = 0.280, 95% CI 0.186–0.420; ≥ RMB 1,500, OR = 0.372, 95% CI 0.221 0.624) and family heredity history of migraine (positive family history, OR = 1.509, 95% CI 1.060 2.148) exerted significant influence on the migraine situation. Specifically, monthly living expenses served as a protective factor, while the family heredity history of migraine was a risk factor. No matter they were associated with migraine separately or integrated, both results were of statistical significance (*P* < 0.05).

**Table 2:** Prevalence of migraine according to participants’ characteristics

Characteristics	*n* (%)	Prevalence (%)	95%Cl (%)	*P*
Total	788	73.20%		
Gender				0.484
Male	166 (21.06%)	71.08	64.12–78.05	
Female	622(78.94%)	73.79	70.33–77.26	
Grade				0.263
Grade 1	419 (53.17%)	72.31	67.51–76.16	
Grade 2	208 (26.40%)	77.40	71.67–83.13	
Grade 3	65 (8.25%)	66.15	54.34–77.97	
Grade 4	96 (12.18%)	74.47	65.49–83.45	
Ethnicity				0.786
Han	721 (91.49%)	73.09	69.85–76.34	
Minority	67 (8.51%)	74.63	63.93–85.32	
History of smoking				0.58
Yes	23 (2.91%)	78.26	60.02–96.50	
No	765 (97.09%)	73.07	69.92–76.22	
History of alcohol consumption				0.564
Yes	39(4.94%)	69.23	54.07–84.39	
No	749(95.06%)	73.35	70.17–76.54	
Living expenses/per month				0.000
≤ 1000¥	128 (16.24%)	50.00	41.22–58.78	
1000¥-1500¥	522 (66.24%)	78.74	75.21–82.26	
≥ 1500¥	138 (17.52%)	73.91	66.50–81.33	
Family history				0.006
Yes	282 (35.78%)	79.08	74.30–83.85	
No	506 (64.22%)	69.96	65.95–73.97	

*n* (%) was the number and percentage for each variable among participants with migraine.

P-values generated from Pearson Chi-square test or Fisher’s exact test where appropriate

**Table 3:** ORs of migraine cases according to characteristics using logistic regression analysis

Characteristics	Crude OR (95% CI)^a^	*P*	Adjusted OR (95% CI)^b^	*P*
Living expense/per month				
≤ 1000	1	0.000	1	
1000–1500	0.270 (0.180–0.405)	0.000	0.280 (0.186–0.420)	0
≥ 1500	0.353 (0.211–0.590)	0.000	0.372 (0.221–0.624)	0
Family history				
No	1		1	
Yes	1.623 (1.150–2.290)	0.000	1.509 (1.060–2.148)	0.022

^a^Only the corresponding independent variable was entered into the model.

^b^The regression model was adjusted for grade, age, body mass index, ethnicity, birthplace, and educational level

### Characteristics of migraine

Table 4 shows the characteristics of migraine. The age of onset for about two-thirds (63.78%) of migraine patients was between 15 and 20 years, and nearly half (49.91%) of migraine patients suffered irregular monthly migraine attacks, which were of statistical significance. More than half (53.38%) of migraine patients had a headache lasting for less than an hour, and very few (3.64%) of them endured more than a 12-h headache. The average scores of male and female were close in VAS.

**Table 4:** Features of migraine and differences between genders

Characteristics	Total *n* = 577	Male *n *= 118	Female *n* = 459	*P*
Age at onset				0.001
< 15 years	201 (34.84%)	51 (43.22%)	150 (32.68%)	
15–20 years	368 (63.78%)	62 (52.54%)	306 (66.67%)	
> 20 years	8 (1.38%)	5 (4.24%)	3 (0.65%)	
Frequency				0.04
Irregular	288 (49.91%)	71 (60.17%)	217 (47.28%)	
Monthly	72 (12.48%)	14 (11.86%)	58 (12.64%)	
Weekly	35 (6.06%)	2 (1.69%)	33 (7.19%)	
1–3 day/week	162 (28.08%)	26 (22.04%)	136 (29.63%)	
4–6 day/week	20 (3.47%)	5 (4.24%)	15 (3.26%)	
Duration				0.513
≤ 1 h	308 (53.38%)	68 (57.63%)	240 (52.29%)	
> 1 h and < 12 h	248 (42.98%)	47(39.83%)	201 (43.79%)	
≥ 12 h	21 (3.64%)	3 (2.54%)	18 (3.92%)	
Visual analog Scale	3.89 ± 1.706	3.84 ± 1.876	3.91 ± 1.661	0.693

Values reported as mean (SD) or n (%) for the total sample, where appropriate.

P-values generated from Mann–Whitney *U* test, Person Chi-square test, or Fisher’s exact test, wherever appropriate

Table 5 presents the common triggers and mitigating factors of migraine. Staying up late was the most common trigger (57.01%), followed by stress (55.29%), cold (54.25%), and sleep problems (52.34%). Emotional changes (43.50%) and fatigue (43.33%) accounted for similar proportions among these triggers. More than one-third of migraine patients (33.45%) caught migraine due to long-term use of electronic products. Sleeping or rest (72.44%) was the most commonly used and the most effective way for themselves to relieve migraine, followed by keeping oneself in a relatively quiet environment (40.37%). More than one-third (37.09%) of patients preferred to mitigate migraine through various forms of massage. Only 139 (24.09%) migraine patients chose to take painkillers to relieve pain.

**Table 5:** Common triggers and mitigating factors of migraine

Trigger factors	*N* (%)	Ranking	Remission factors	*N* (%)	Ranking
Staying up late	329 (57.01)	1	Sleeping or rest	418 (72.44)	1
Stress	319 (55.29)	2	Quiet environment	233 (40.38)	2
Catch a cold	313 (54.25)	3	Massage	214 (37.09)	3
Sleeping problems	302 (52.34)	4	Take painkiller	139 (24.09)	4
Emotional changes	251 (43.50)	5	Medical care	120 (20.79)	5
Fatigue	250 (43.33)	6	Heat Application	46 (7.97)	6
Long-term use of electronic products	193 (33.45)	7	Cold Application	19 (3.29)	7
Frosty winds	184 (31.89)	8	Wear a hat	13 (2.25)	8
Taking public transportation	180 (31.20)	9			
Heat	151 (26.17)	10			
Chill	142 (24.61)	11			
Weather change	140 (24.26)	12			
Noise	119 (20.62)	13			
Irritability smell	75 (12.99)	14			
Sunlight	57 (9.88)	15			
Exercises	57 (9.88)	15			
Season change	57 (9.88)	15			
Alcohol	53 (9.19)	16			
Smoking	19 (3.29)	17			
Coffee	13 (2.25)	18			

### Headache locations

The nature of migraine pain is mainly dominated by unilateral and paroxysmal moderate–severe pulsatile pain [8, 27]. Table 6 shows the gender ratio of unilateral and bilateral headache. The results reveal that the participants were more plagued by bilateral headache than by unilateral headache under the same gender.

**Table 6:** Headache locations of migraine patients based on gender, unilateral headache, and bilateral headache

Gender	One side	Both side
Male	34 (47.89%)	37 (52.11%)
Female	174 (43.61%)	225 (56.39%)

### Accompanying symptoms

Previous studies have demonstrated that migraine attacks are often accompanied by nausea, vomiting, photophobia, phonophobia, and other symptoms [8, 13, 27, 28]. Our study also reveals that nausea, photophobia, phonophobia, and vomiting are the common accompanying symptoms, ranking the second, the fourth, the fifth, and the sixth, respectively. More than half (50.43%) of migraine patients were reported to have dizziness during a migraine attack, and more than one-third (33.45%) stated that vertigo was a typical accompanying symptom (Table 7).

**Table 7:** Accompanying symptoms of migraine patients and their ranking

Accompanying symptoms	*N* (%)	Ranking
Dizziness	291 (50.43)	1
Nausea	242 (41.94)	2
Vertigo	193 (33.45)	3
Photophobia	134 (23.22)	4
Phonophobia	121 (20.97)	5
Vomiting	115 (19.93)	6
Abdominal discomfort	67 (11.61)	7
Visual symptoms	47 (8.15)	8
None	40 (6.93)	9
Aversion irritability smell	19 (3.29)	10

## Discussion

Among the 9057 college students in our cluster sampling, 8,783 passed the preliminary screening of the ID-Migraine questionnaire. Based on the final screening results, 577 (55.64%) out of 1,037 college students were diagnosed with migraine, and 211 (20.35%) with tension headache and other types of headache. The remaining patients with positive results during the preliminary screening cannot be diagnosed with migraine based on clinical evidence and ICHD-3 due to the short duration and mild symptoms of the first migraine, and some had a secondary headache or other types of headache that can not be classified by ICHD-3. Our study found that migraine is very common among medical students in North Sichuan Medical College, affecting 6.57% of the population. The incidences of students of the female gender, lower grades, or with family heredity history of migraine are significantly higher than those of other groups. The onset age of most (63.78%) migraine patients is within 15–20 years, and the headache duration of patients with mild symptoms is not long. In addition, staying up late, stress, cold and sleep disorders remain common triggers.

In our study, the overall migraine incidence of medical students in North Sichuan Medical College is slightly lower than that in medical schools of northern China (8.6%) [2] and Soochow University (7.91%) [12], which is possibly due to low temperature in Northern China as the climate itself is one of the triggers of migraine. It also indicates that the incidence of migraine may be somewhat related to geographical location. Compared with overseas universities, our prevalence rate is far lower than that of the United States (24.8%) [29] and that of Kuwait University (27.86%) [4], similar to that of southeastern Iran (7.14%) [4], but higher than that of Nigeria (6.4%) [30]. Compared with the overall average, our prevalence rate is lower than that of the global population (10–18%) [1], that of all college students (16.1%) [18], and that of medical students from medical schools (11–40%) [19].

Some of the existing studies generally showed that the prevalence rate of migraine in women was higher than that in men [7, 14, 30, 31], and our results also showed a good agreement (6.77% > 5.90%), which may be related to the hormone level in women's menstrual cycle, leading to easier induction and longer duration of migraine [32]. At the same time, some studies have shown that there was no significant difference in the prevalence rates between men and women [2]. According to our results, among 788 participants with headaches, the incidence rate of patients with a family heredity history of headache was 79.1% (95%CI 74.3–83.9), and *P* = 0.006 < 0.05, which was statistically significant. Among 577 college students diagnosed with migraine, 223 (38.65%) were found with family heredity history, indicating that genetic predisposition played an important role in the occurrence of migraine.

Our study showed that the prevalence rate was higher among students in grade one and grade two (8.01% and 8.05%), which might be attributed to the change in the surrounding environment. Lower-grade students are still adapting to their college life, and the different learning modes from high school and the new learning pressure lead to the high incidence of migraine in them. Compared with lower grade students, higher grade students have adapted to the learning mode in universities and could better cope with heavy academic pressure and stressful final exams, so they had a relatively low prevalence rate. However, some studies have shown that the occurrence of migraine was irrelevant to grade level [30, 33], which could be explained by two possible reasons: 1. due to the difference in course schedule in each grade, the learning length and learning pressure of students also vary with grade. 2. Some students involved in the study may be at the beginning of a semester, while others may be at the end of the semester and are under the pressure of final exams, thus leading to the consequence that these results lack statistical significance.

Compared with the general population suffering from unilateral pain, most patients in our research are suffering from bilateral pain, which is possibly due to the following reasons: 1. the symptoms of the first-episode migraineurs are not the same as those of the typical migraineurs, and differences may also occur in the location and nature of pain as well as in other characteristics of pain attack. 2. It is worth further exploring whether these differences can only be found among the university students pursuing a medical degree, to provide the basis for the diagnosis of first migraine or migraine with atypical symptoms in the future.

We found that in addition to suffering from the typical accompanying symptoms of nausea, vomiting, photophobia, and phonophobia, a high proportion of patients always suffer from dizziness (50.43%) and vertigo (33.45%). Among all types of migraine, vertigo and vestibular migraine are closely linked [34, 35], and some symptoms of vestibular migraine can also be manifested as dizziness. Therefore, if the subtypes of migraine are further distinguished, different common accompanying symptoms may occur in different types of migraine, which is also worthy of our further analysis. Studies in this respect will assist in the diagnosis and precise treatment of migraines in the future.

At the same time, our research also led to an interesting discovery. Table 2 shows that the amount of monthly living allowance (*P* = 0.000 < 0.05) and family history of migraine (*P *= 0.006 < 0.05) are closely related to migraine attacks. Including these two factors in the further logistic studies (Table 3), it is found that the family history of migraine is a risk factor, which has been confirmed in many studies [4, 12, 20]. In addition, the economic factor is also an important trigger for migraine attacks [36]. Our research results show that the monthly living allowance is a protective factor, but the protective effect will not rise with the increase of the living allowance. When it is at the intermediate level (1000 ¥ —1500 ¥), the protective effect is the strongest (OR = 0.280 < 0.372). This finding indicates that an optimal standard of monthly living allowance can be found to minimize the risk of migraine attacks. However, due to the different economic development, consumption level, races, environment, and other factors in a different area, the standard may not be unified. But if the research only targets the level of living allowance in some certain area, the discovery of an optimal parameter may seem more possible.

According to the research conducted in the United States [29], Kuwait [4], Nigeria [30], Brazil [14], and other places, the degree of migraine pain often ranges between being moderate and severe. However, our research shows that the degree of migraine pain for most medical students was between being mild and moderate, and the overall average VAS score was 3.89 ± 1.706, which is similar to the study results by Soochow University [12]. The connection between the degree of migraine pain and the ethnic groups is worth further exploration. Meanwhile, it was found that nearly half of the students (49.91%) suffered from irregular migraine attacks, more than half of the students (53.38%) suffered from migraine attacks lasting less than 1 h, and only 21 students (3.64%) suffered from migraine attacks lasting more than 12 h.

Based on our research, lack of sleep (staying up late), poor sleep quality, and stress are common triggers. These findings are consistent with the results of the research conducted by the University of Kuwait [4], the University of Gondar in Ethiopia [37], or in the United States [29] and Brazil [14]. Most medical students around the world suffer from sleep disturbance. More interestingly, it was found that catching a cold is also a common trigger very possibly leading to migraine attacks (accounting for 54.25% of migraine attacks). It is listed as the third most common trigger, followed by sleep disorders (accounting for 52.34% of migraine attacks). Patients involved in the research investigation said that cold symptoms were often accompanied by migraine attacks, which was rarely mentioned in other studies. We deem that the possible reason is that due to academic pressure and clinical work at the hospital, students have little free time to relax and lack in physical exercises. At the same time, staying up late for a long time and poor sleep quality aggravate the burden of their body. All these factors form a vicious circle and result in the decline of their physical resistance. What makes things worse is that some academic tasks have to be completed in the hospital, a more susceptible environment. Therefore, students are more likely to catch a cold in that environment, thus triggering migraine attacks.

Suffering from migraine attacks, the vast majority of them (72.44%) chose to relieve the headache by having more sleep and rest. Only 139 patients (24.09%) chose to take painkillers, and 120 patients (20.79%) seek medical treatment under the guidance of doctors. This proportion of patients seeking medical help is far lower than that of the general people with migraine in the United States [38] and China [39]. We deem that the possible reasons are as follows: 1. due to the busy course schedule and stressful clinical work, students lack time for medical treatment. When migraine attacks, they often choose to take medicine according to their medical knowledge and experience and refuse to see a doctor. 2. Some patients pay little attention to the early attacks of migraine, or they do not know that their physical discomfort is actually caused by migraine, but mistake it as a cold or other diseases. Since the symptoms are mild, they choose to relieve the symptoms by sleeping, resting or in other ways rather than receiving regular medical treatment. 3. According to our statistics, the headache duration for most patients is short (< 1 h, *n* = 308, 53.38%), which may be one of the reasons for their refusing to see a doctor.

More considerations should be given to relieve the students’ pressure from academic work and reduce their working hours simply and effectively and to solve their sleep problems by asking them to set more reasonable course schedules and academic arrangements and spend more time on physical exercises and extracurricular activities. It is of great value to the prevention and treatment of migraine by completing all the above work. At the same time, when migraine attacks, students should receive the correct guidance as to how to relieve their headache and seek medical treatment more rationally and effectively, and how to avoid drug abuse, drug overuse, and other problems.

## Limitations

In our research, a scientific preliminary screening was conducted among 9057 students from medical colleges and universities in China through cluster sampling by using ID-Migraine. Such a large scale of preliminary screening and the huge size of sampling are not available in other areas of China. In the end, 577 patients were diagnosed with migraine. The analysis of triggers and headache conditions for 577 patients among the samples was representative. However, there are still some limitations in our research as follows. Firstly, our research was only conducted among medical students and did not include non-medical students for comparison, which may lead to a lack of sampling representativeness in the results. Second, as our study was a cross-sectional study, some degree of recall bias might occur in the survey among patients with headache. Third, different subtypes of migraine were not compared. Fourth, it is a pity secondary headache was not included in our research, as the comparison of secondary headache and primary headache may contribute to the clinical diagnosis and treatment.

## Conclusion

Migraine is common among medical students in North Sichuan Medical College, and even more common among female students, junior university students, and students with a family history of migraine. Staying up late, stress, influenza, and sleep disorders are the most common triggers. In future studies, the focus should be given to the prevention and treatment of various subtypes of migraine. Medical colleges and universities should offer relevant courses on the prevention and treatment of migraine as well as the intervention and treatment of this disease during migraine attacks, to enable medical students to have a deeper understanding of it and relieve the pain caused by migraine more effectively.

## Data Availability

The datasets used and/or analyzed during the current study are available from the corresponding author on reasonable request.
